# Antinociceptive effects of morphine and naloxone in mu-opioid receptor knockout mice transfected with the MORS196A gene

**DOI:** 10.1186/1423-0127-17-28

**Published:** 2010-04-20

**Authors:** Shiou-Lan Chen, Hsin-I Ma, Jun-Ming Han, Ru-Band Lu, Pao-Luh Tao, Ping-Yee Law, Horace H Loh

**Affiliations:** 1 Department of Psychiatry, College of Medicine and Hospital, National Cheng Kung University, Tainan, Taiwan; 2 Department of Neurosurgery, Tri-Service General Hospital, Taipei, Taiwan; 3 Department of Pharmacology, National Defense Medical Center, Taipei, Taiwan; 4 Department of Pharmacology, University of Minnesota, Minneapolis, Minnesota, USA

## Abstract

**Background:**

Opioid analgesics such as morphine and meperidine have been used to control moderate to severe pain for many years. However, these opioids have many side effects, including the development of tolerance and dependence after long-term use, which has limited their clinical use. We previously reported that mutations in the mu-opioid receptors (MOR) S196L and S196A rendered them responsive to the opioid antagonist naloxone without altering the agonist phenotype. In MORS196A knock-in mice, naloxone and naltrexone were antinociceptive but did not cause tolerance or physical dependence. In this study we delivery this mutated MOR gene into pain related pathway to confirm the possibility of *in vivo *transfecting MORS196A gene and using naloxone as a new analgesic agent.

**Methods:**

The MOR-knockout (MOR-KO) mice were used to investigate whether morphine and naloxone could show antinociceptive effects when MORS196A gene was transfected into the spinal cords of MOR-KO mice. Double-stranded adeno-associated virus type 2 (dsAAV2) was used to deliver the MORS196A-enhanced green fluorescence protein (EGFP) gene by microinjected the virus into the spinal cord (S2/S3) dorsal horn region. Tail-flick test was used to measure the antinociceptive effect of drugs.

**Results:**

Morphine (10 mg/kg, s.c.) and naloxone (10 mg/kg, s.c.) had no antinociceptive effects in MOR-KO mice before gene transfection. However, two or three weeks after the MOR-S196A gene had been injected locally into the spinal cord of MOR-KO mice, significant antinociceptive effects could be induced by naloxone or morphine. On the other hand, only morphine but not naloxone induced significant tolerance after sub-chronic treatment.

**Conclusion:**

Transfecting the MORS196A gene into the spinal cord and systemically administering naloxone in MOR-KO mice activated the exogenously delivered mutant MOR and provided antinociceptive effect without causing tolerance. Since naloxone will not activate natural MOR in normal animals or humans, it is expected to produce fewer side effects and less tolerance and dependence than traditional opioid agonists do.

## Background

Morphine, which acts primarily on the mu-opioid receptors (MOR), is used clinically to control moderate and severe pain. However, morphine has many adverse side effects, such as respiratory depression, vomiting, nausea, constipation, tolerance, and dependence. After prolonged use, analgesic tolerance develops, which requires dosage increases to maintain its analgesic effect. This is problematic because dosage increases also increase the frequency and severity of its side effects. Therefore, developing new analgesics without these side effects is imperative.

We previously created MORS196A, a single-point mutation of Ser-196 in the fourth transmembrane domain of the MOR to Ala [1]. MORS196A recognizes the opioid antagonist naloxone and naltrexone as partial agonists. In Chinese hamster ovary cells stably expressing the S196A mutant, naloxone and naltrexone inhibited forskolin-stimulated adenylyl cyclase activity. Antagonists also activated the G-protein-coupled inwardly rectifying potassium channel 1 (GIRK1) in *Xenopus *oocytes co-expressing the S196A mutant and the GIRK1 channel. The ability of opioid antagonists to activate MORS196A *in vitro *was also shown *in vivo *[2],. Morphine was equally antinociceptive in homozygous MORS196A knock-in mice and in wild-type mice. Naloxone and naltrexone were also antinociceptive but did not cause tolerance or physical dependence in the MORS196A knock-in mice [2]. We recently [3] investigated the efficiency of using double-stranded adeno-associated virus type 2 (dsAAV2) vectors to deliver the MORS196A-enhanced green fluorescence protein (EGFP) gene into the sacral spinal cord of healthy wild type Imprinting Control Region (ICR) mice. We found that a single injection provided sustained gene expression in the spinal cord for at least 6 months [3]. In ICR mice expressing the mutant MOR, morphine induced similar antinociceptive responses and induced tolerance and withdrawal symptoms and reward effects similar to those in control mice (only saline injected into the spinal cord). Conversely, in ICR mice injected with the mutant MOR gene, naloxone also had antinociceptive effect, but it had no measurable effect in control mice. Furthermore, the chronic administration of naloxone to mice expressing the mutant MOR did not induce tolerance, dependence, or reward responses. Because of the wide distribution of non-mutated MOR in these ICR mice, we have used MOR-KO mice to further confirm our findings in the present study.

## Methods

### Chemicals

Morphine hydrochloride was purchased from the National Bureau of Controlled Drugs, National Health Administration, Taipei, Taiwan. Naloxone and naltrexone were purchased from Sigma-Aldrich Chemical Co. (St. Louis, MO, USA). All other chemicals were locally purchased and of analytical grade.

### Constructing AAV2-MORS196A-EGFP plasmid

DsAAV-MORS196A-EGFP plasmid was constructed by replacing the GFP gene and the SV40 polyA site of the dsAAV-CMV-EGFP gene with MORS196A-EGFP cDNA at the BamHI and NotI sites, coupled to a miniature polyA site. The recombinant viral stocks were produced by the adenovirus-free, triple-plasmid cotransfection method [4]. The AAV vectors were purified by double CsCl centrifugation and the titers were determined by dot blot assay in the range of 1.0-3 × 10^13 ^viral particles/ml.

### Experimental Animals

Twelve MOR-KO mice were kindly provided by Dr. Horace H. Loh [5]. The mice were housed in a room with a 12/12-h light/dark cycle, at a temperature of 25 ± 2°C and a humidity of 55%. A standard rodent diet and water were provided ad libitum. The care of animals was carried out in accordance with institutional and international standards (Principles of Laboratory Animal Care, National Institutes of Health), and the protocol had the approval of the Institutional Animal Care and Use Committee of the National Defense Medical Center (Taiwan, Republic of China).

### Direct microinjection of dsAAV vectors into the spinal cord dorsal horn

The mice were intraperitoneally (i.p.) injected with pentobarbital (100 mg/kg), put under a dissecting microscope, and then given a partial dorsal laminectomy. One of the lumbar processes at L1-L2 was carefully removed to expose a segment of spinal cord (S2-S3). Each mouse was then placed in a spinal frame holder and mounted under a stereotaxic frame with a microinjector attachment that included a 10-μl Hamilton syringe with a micro-tipped glass pipette. Four doses (0.5 μl) of the dsAAV2-MORS196A-EGFP were bilaterally injected into the spinal cord dorsal horn at a depth of approximately 0.3 mm. After surgery, the muscle and skin around the wound were sutured and the wounds were held together with three microsurgical wound clips.

### Determining the antinociceptive effect of the drugs

**Figure 1:**
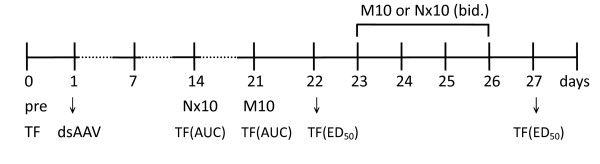
**The experimental schedule of MOR-KO mice**. Before microinjection of the dsAAV vectors (pre; 0 day), the antinociceptive effects of saline, morphine (M10; 10 mg/kg, s.c.), and naloxone (Nx10; 10 mg/kg, s.c.) were tested by tail-flick test (TF) as the pretest data. After dsAAV injections, the mice (n = 12) were tested with either Nx10 (s.c., on 14^th ^day) or M10 (s.c., on 21^th ^day). The AUC values of tail-flick test were calculated respectively. Mice were then separated into 2 groups (n = 6) and injected with either M10 (s.c., b.i.d) or Nx10 (s.c., b.i.d.) for 4 days. The ED_50 _of the drugs were evaluated before and after the sub-chronic drugs treatment.

Drug-induced antinociceptive effect was evaluated using the tail-flick test. Using a tail-flick apparatus (Model 37360; Ugo Basile, Comerio VA, Italy), the intensity of the heat source was set at 35, which allowed the basal tail-flick latency to be controlled between 3 and 4.5 s for all mice (cut-off time: 10 s). The area under the curve (AUC) from the time-response curve or ED_50 _value was considered an index for the antinociceptive effect of the drugs. Tail-flick latency was recorded at 30, 60, 90, 120, and 180 min after the drug had been injected. The AUC value was obtained by calculating the area under the time-response curve of the antinociceptive effect (test latency - basal latency) from 0 to 180 min after the administration of the drugs. The ED_50 _was determined using the up-and-down method described by Dixon [6]. As shown in Fig. 1, before microinjection of the vectors (pre, 0 day), the antinociceptive effects of saline, morphine (M10; 10 mg/kg, s.c.), and naloxone (Nx10; 10 mg/kg, s.c.) were tested as the pretest data. From two to three weeks after the dsAAV injection, the mice (n = 12) were tested with either naloxone (10 mg/kg, s.c. on 14^th ^day) or morphine (10 mg/kg, s.c. on 21^th ^day). The AUC values of tail-flick test were calculated respectively. To test drug-induced tolerance, the mice were separated into 2 groups (n = 6) and injected with either morphine (10 mg/kg, s.c.) or naloxone (10 mg/kg, s.c.) twice a day (b.i.d.) for 4 days. The ED_50 _of the drugs were evaluated before and after the sub-chronic drugs treatment.

### Fluorescence microscopy

The mice were anesthetized with pentobarbital (100 mg/kg, i.p.) and transcardially perfused with Tyrone's calcium-free buffer (116 mM NaCl, 5.36 mM KCl, 1.57 mM MgCl_2_.6H_2_O, 0.405 mM MgSO_4_, 1.23 mM NaH_2_PO_4_, 5.55 mM Glucose, 26.2 mM NaHCO_3_, pH 7.4), and then with 4% paraformaldehyde in 0.1 M phosphate buffer. The sacral spinal cord was dissected and placed in 20% sucrose solution at 4°C overnight. The samples were then embedded in OCT compound and immediately frozen at -80°C. Serial transverse spinal cord slices (10 μgm) were sectioned using a cryostat. The slices were mounted on slides (Super Frost Plus; Menzel-Glaser, Braunschweig, Germany) and a fluorescence microscope was used to visualize the green fluorescence which represented the dsAAV-mediated transgenic expression of MORS196A.

### Statistical analysis

Data are means ± the standard error of the mean (SEM). One-way analysis of variance (ANOVA) and the Newman-Keuls test were used to analyze and compare the data. Statistical significance was set at *P *< 0.05.

## Results

### DsAAV2-MORS196A-EGFP gene transfection and expression *in vitro *and *in vivo*

**Figure 2:**
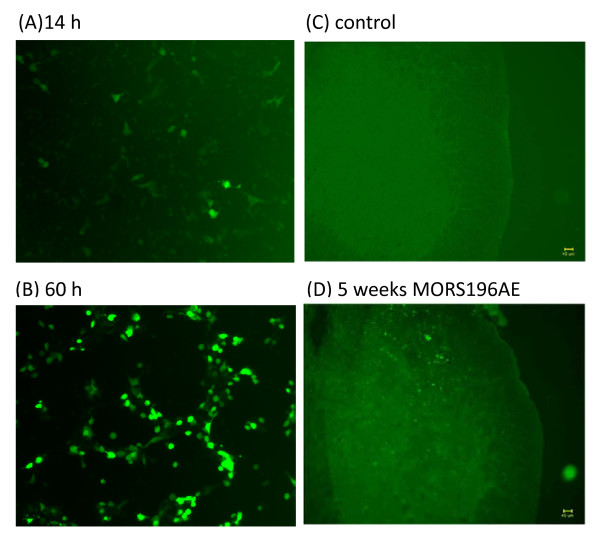
**Fluorescence micrographs *in vitro *and *in vivo*.** (A)-(B) Representative fluorescence micrographs of HEK-293 cells 14-60 h after they had been transfected with the dsAAV2-MORS196A-EGFP gene (magnification: 100×). (C) A representative spinal cord slice from a control mouse not transfected. (D) A representative spinal cord slice from MOR-KO mice four weeks after transfection (magnification: 100×). Scale bars = 40 μm.

HEK-293 cells expressed the MORS196A-EGFP substantially more 60 h (Fig. 2B) than 14 h (Fig. 2A) post-transfection. The dsAAV-MORS196A-EGFP virus was then harvested and purified. The viral titer used in this experiment was 1 × 10^13 ^vp/ml. To observe the expression of the mutant receptor, the spinal cords of the mice were removed and sectioned at 4^th ^week. Significant green fluorescence that represented the expression of MORS196A could be observed in the dorsal horn of sacral spinal cord (Fig. 2D). In contrast, no significant green fluorescence could be observed in the spinal cord of control mice (Fig. 2C).

### Morphine was antinociceptive and induced tolerance after the transfection of the dsAAV2-MORS196A-EGFP gene

**Figure 3:**
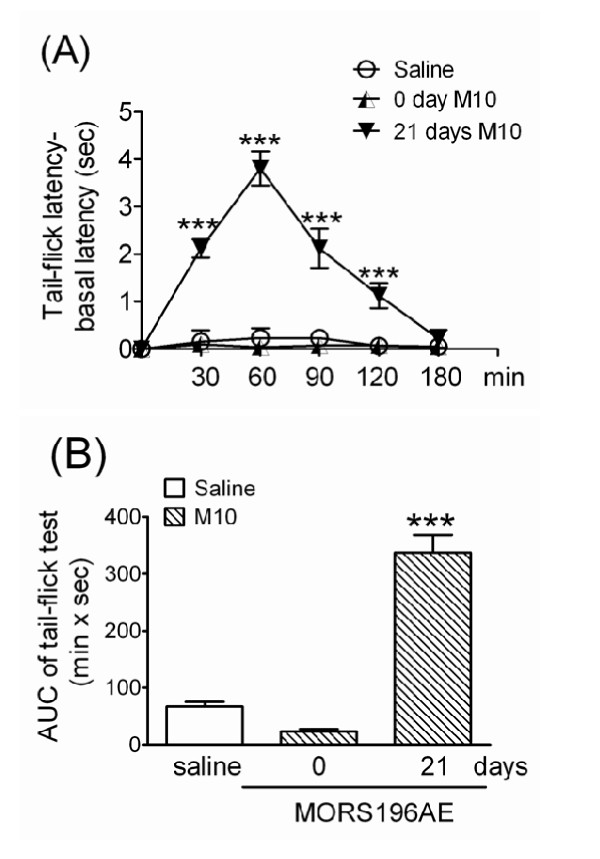
**Antinociceptive effect of morphine in MOR-KO mice determined using tail-flick tests before and after they had been transfected with the dsAAV2-MORS196A-EGFP gene**. (A) The time-response curves for saline and 10 mg/kg of morphine before (0 day M10) and 21 days (21 days M10) after transfection. ***P < 0.001 (vs. saline). (B) The mean AUC (area under curve) values for each treatment in Fig. 3A (n = 12). ***P < 0.001 (vs. saline).

**Figure 4:**
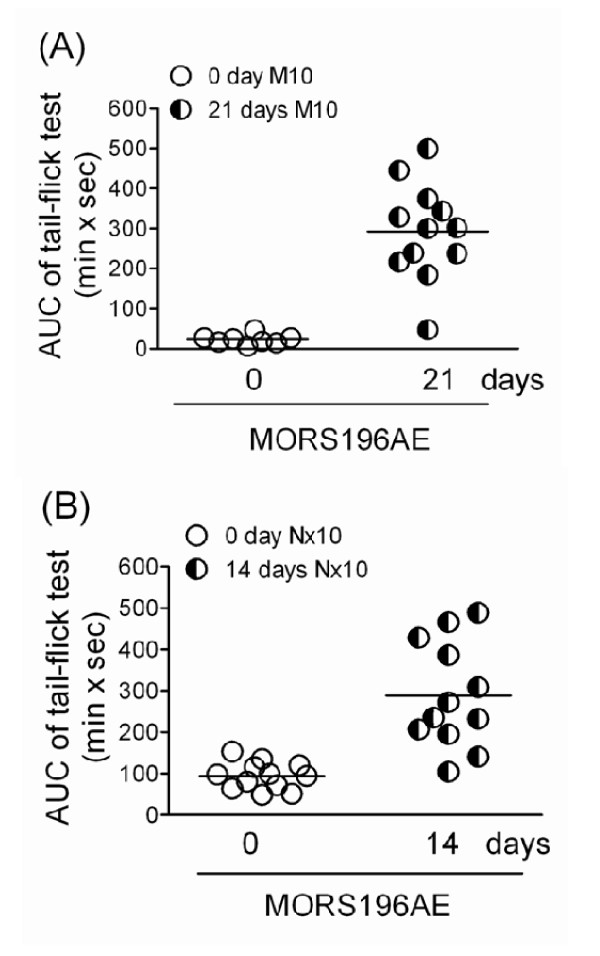
**Antinociceptive effects of morphine and naloxone in individual MOR-KO mice before and after they had been transfected with the dsAAV2-MORS196A-EGFP gene**. (A) The AUC (area under curve) values of morphine 10 mg/kg (s.c.) before (0 day M10) and 21 days (21 days M10) after transfection. (B) The AUC values of 10 mg/kg of naloxone (Nx10, s.c.) before (0 day Nx10) and 14 days (14 days Nx10) after transfection.

As expected, morphine (10 mg/kg, s.c.) did not have antinociceptive effect in MOR-KO mice before transfection of the dsAAV2-MORS196A-EGFP gene (Fig. 3A, 0 day M10). However, 21 days after transfection, morphine (10 mg/kg. s.c.) showed significant antinociceptive effect (Fig. 3A, 21 days M10). The AUC value of each mouse was calculated and shown in Fig 4A. The mean AUC value of the tail-flick test was 337.6 ± 51.7 (min × sec) 21 days post-transfection (Fig. 3B). The mice were then treated with morphine (10 mg/kg, b.i.d., s.c.) for 4 days, and the ED_50 _increased from 4.4 ± 1.0 to 7.2 ± 1.1 mg/kg, which indicated the development of approximately 1.63 fold degree of tolerance (Table 1).

**Table 1:** Tolerance induced by morphine but not naloxone in MOR-KO mice that had been transfected with the dsAAV2-MORS196A-EGFP gene

	ED_50 _of morphine (mg/kg)	ED_50 _of naloxone (mg/kg)
Acute effect	4.4 ± 1.0	8.4 ± 1.2

Sub-chronic effect	7.2 ± 1.1 (×1.63)	8.4 ± 1.2

The ED_50 _values of morphine and naloxone that inhibit the tail-flick responses were determined using the up-and-down method 3 weeks after gene transfer. The mice were then sub-chronically treated with morphine (10 mg/kg) or naloxone (10 mg/kg) subcutaneously twice a day for 4 days. ED_50 _values were then redetermined. Data are means ± the standard error of the mean (SEM) (*n *= 6).

### Naloxone elicited antinociceptive effects without tolerance in mice transfected with dsAAV2-MORS196A-EGFP gene

**Figure 5:**
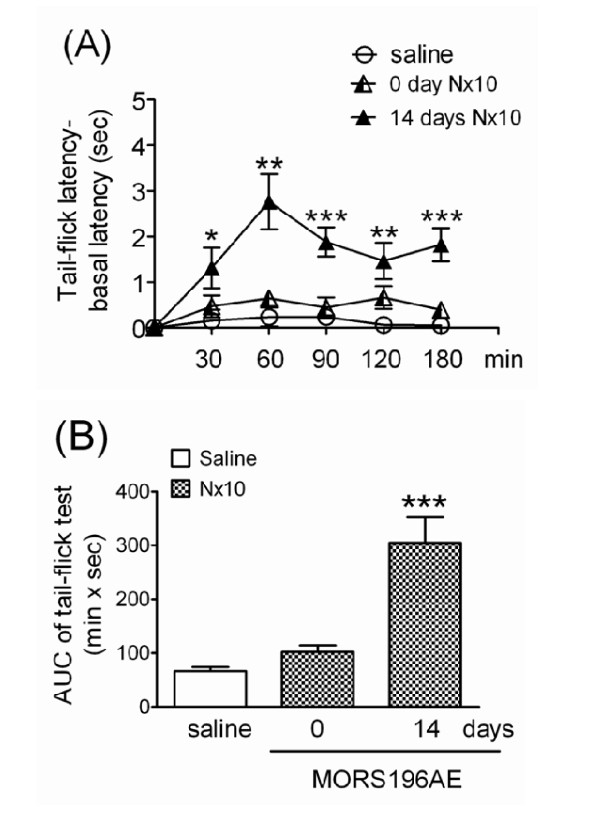
**Antinociceptive effect of naloxone in MOR-KO mice determined using tail-flick tests before and after they had been transfected with the dsAAV2-MORS196A-EGFP gene**. (A) The time-response curves for saline and 10 mg/kg naloxone before (0 day Nx10) and 14 days (14 days Nx10) after transfection. *P < 0.05, **P < 0.01, ***P < 0.001 (vs. saline). (B) The mean AUC (area under curve) values for each treatment in Fig. 5A (n = 12). ***P < 0.001 (vs. saline).

Naloxone (10 mg/kg, s.c.) also did not show antinociceptive effect in MOR-KO mice before transfection of the dsAAV2-MORS196A-EGFP gene. The mean AUC value of 10 mg/kg of naloxone [94.3 ± 9.5 (min × sec)] was not significantly different from that of saline [66.3 ± 7.9 (min × sec)] before transfection (Fig. 5). However, significant antinociceptive effects were presented from 30 min to 180 min after naloxone was administered subcutaneously 14 days after transfection (Fig. 5A, 14 days Nx10). The AUC value of each mouse was calculated and shown in Fig 4B. The mean AUC value of the same dose of naloxone significantly increased to 304.7 ± 47.4 (min × sec) 14 days after transfection (Fig. 5B). The ED_50 _of naloxone (8.4 ± 1.2 mg/kg) did not change after 4 days of sub-chronic treatment with naloxone (10 mg/kg, b.i.d., s.c.), which indicated that there was no tolerance developed to naloxone (Table 1).

## Discussion

In this study, double-strand AAV2 was chosen for neuronal expression of the mutant MORS196A receptor. Its extensive transduction into neurons and long-term gene expression with no apparent toxicity made this recombined virus as a potential candidate vector for gene therapy [7]. The double-strand AAV2 vector had been demonstrated the efficiency of transferring the target gene to the central nervous system [8-10]. After single injection of MORS196A gene, the local and sustained MORS196A receptor expression in dorsal horn of sacral to lumbar spinal cord were found in these MOR knock-out mice. Our findings further confirm the gene transfer efficiency of recombined AAV2 in central nervous system.

In the present study we only got totally 12 male MOR knock-out mice. Therefore we have tried to examine the effect of morphine at a dose of 5 mg/kg (s.c.) on 7^th ^day initially and found morphine had already shown significant antinociceptive effect (data not shown). Later on 14^th ^day and 21^th ^day, the effects of naloxone (s.c.) or morphine at a dose of 10 mg/kg (s.c.) were determined separately in all 12 mice. In order not to confuse the reader, we have deleted the data of morphine at a dose of 5 mg/kg (s.c.) on 7^th ^day and compared the pretest data of morphine (10 mg/kg, s.c.) with the same dose of morphine on 21^th ^day post-transfection (21 days M10) (Fig 3). We found that morphine at a dose of 10 mg/kg did not have significant antinociceptive effect in MOR-KO mice before they were transfected with the MORS196A-EGFP gene. Previous studies also reported that morphine at doses up to 56 mg/kg did not show significant antinociceptive effect in homozygous MOR-KO mice [11,12,5]. However, after 21 days transfection of MORS196A-EGFP gene, 10 mg/kg (s.c.) morphine showed significant antinociceptive effect. We also found that 1.63 fold of tolerance developed after the transfected MOR-KO mice had been sub-chronically treated with morphine (10 mg/kg, s.c., b.i.d.) for 4 days. These results resembled the previous MORS196A knock-in mice study, morphine could elicit the antinociceptive effect and induced tolerance [2]. Our findings indicate that morphine can activate the exogenously delivered and expressed mutant MOR receptor in spinal cord and induces tolerance in MOR-KO mice. On the other hand, naloxone, an opioid receptor antagonist, was also shown significant antinociceptive effect but did not cause tolerance in this local MORS196A gene transfer MOR-KO mice. Previous reports have indicated that the delta opioid receptor (DOR) plays an essential role in the formation of opioid tolerance [13]. Co-administration of morphine with delta opioid receptor (DOR) antagonists, thus inactivating the DORs, blocks morphine tolerance in mice [13]. The loss of morphine tolerance in DOR-KO mice also had been documented [14]. Therefore, it is reasonable to surmise that an opioid antagonist (naloxone) that activates an exogenously delivered mutant MOR receptor and inactivates the endogenous DOR will inhibit the development of tolerance to that opioid. The present data further support our previous findings in ICR mice [3] and suggest that, if the mutant opioid receptor can be delivered to the pain related pathway, opioid antagonist mediated activation of this mutant receptor should result in pain relieve without the development of tolerance.

We also found that not all of the transfected MOR-KO mice responded to morphine and naloxone treatment. Although every mouse showed the EGFP expression in the sacral and lumbar spinal cord, only 9 of 12 (75%) mice responded significantly to the naloxone or morphine treatment (AUC > 200 min × sec, Fig 4). Except the individual variation, the possible reason for this may be that MORS196A-EGFP could express not only at the primary sensory neuron, but also at many other neurons that might not be related to the antinociception. For achievement of the ideal antinociception, the neuron specific promoter or vector must be developed for the more efficient gene transfer in future study.

Our results provided a possibility of dsAAV2 mediated local gene transfer and use of opioid antagonists to treat chronic pain without tolerance in future clinic study. However, in the present study, we have used the direct local injection of vector into the local site of spinal cord. Although spinal-cord microinjection was convenient for the present study, it is not ideal for human therapy. Intrathecal transfection into the subarachnoid space of the spine will be less invasive and more patient-friendly. Moreover, morphine-dependent patients may undergo withdrawal symptoms when they begin naloxone treatment. Therefore, a carefully designed treatment must be developed. A local intrathecal delivery of the mutant MOR and gradual weaning from morphine and then using naloxone may be ideal strategies for pain therapy in morphine-dependent patients. To achieve this goal, more studies on the safety and the efficiency of dsAAV mediated local gene transfer still need carefully design and monitor in future study.

## Conclusion

DsAAV2 efficiently delivered the target gene (MORS196A) to the spinal cord in MOR-KO mice. *In vivo *transfecting the MORS196A gene into the pain related nervous pathway and systemically administering naloxone activated the local expressed mutant MOR and induced antinociceptive effect without tolerance. This treatment may become an alternative to traditional opioid agonists for pain management.

## Abbreviations

AUC: area under the curve; DOR: delta opioid receptor; dsAAV2: double-stranded adeno-associated virus type 2; EGFP: enhanced green fluorescence protein; ICR: Imprinting Control Region; MOR: mu-opioid receptors; MOR-KO: MOR-knockout.

## Competing interests

The authors declare that they have no competing interests.

## Authors' contributions

PLT, PYL and HHL designed research. SLC carried out all the animal experiments. JMH and HIM worked on the production of the viral vectors. The manuscript was drafted by PLT, SLC and RBL All authors read and approved the final manuscript.
